# Heat Stress, Physiological Response, and Heat-Related Symptoms among Thai Sugarcane Workers

**DOI:** 10.3390/ijerph17176363

**Published:** 2020-09-01

**Authors:** Pongsit Boonruksa, Thatkhwan Maturachon, Pornpimol Kongtip, Susan Woskie

**Affiliations:** 1 School of Occupational Health and Safety, Institute of Public Health, Suranaree University of Technology, 111 University Ave., Muang, Nakhon Ratchasima 30000, Thailand; 2 School of Community Health Nursing, Institute of Nursing, Suranaree University of Technology, 111 University Ave., Muang, Nakhon Ratchasima 30000, Thailand; thatkhwan@sut.ac.th; 3 Department of Occupational Health and Safety, Faculty of Public Health, Mahidol University, 420/1 Rajvithi Road, Bangkok 10400, Thailand; pornpimol.kon@mahidol.ac.th; 4 Center of Excellence on Environmental Health and Toxicology, EHT, Bangkok 10400, Thailand; 5 Department of Public Health, University of Massachusetts Lowell, One University Ave, Lowell, MA 01854-2867, USA; Susan_Woskie@uml.edu

**Keywords:** heat stress, wet bulb globe temperature (WBGT), heat-related symptom, heat strain, sugarcane worker, harvesting season

## Abstract

Prolonged or intense exposure to heat can lead to a range of health effects. This study investigated heat exposure and heat-related symptoms which sugarcane workers (90 sugarcane cutters and 93 factory workers) experienced during a harvesting season in Thailand. During the hottest month of harvesting season, wet bulb globe temperature was collected in the work environment, and workloads observed, to assess heat stress. Urine samples for dehydration test, blood pressure, heart rate, and body temperature were measured pre- and post-shift to measure heat strain. Fluid intake and heat-related symptoms which subjects had experienced during the harvesting season were gathered via interviews at the end of the season. From the results, sugarcane cutters showed high risk for heat stress and strain, unlike factory workers who had low risk based on the American Conference of Governmental Industrial Hygiene (ACGIH) threshold limit values (TLVs) for heat stress. Dehydration was observed among sugarcane cutters and significant physiological changes including heart rate, body temperature, and systolic blood pressure occurred across the work shift. Significantly more sugarcane cutters reported experiencing heat-related symptoms including weakness/fatigue, heavy sweating, headache, rash, muscle cramp, dry mouth, dizziness, fever, dry/cracking skin, and swelling, compared to sugarcane factory workers. We conclude that the heat stress experienced by sugarcane cutters working in extremely hot environments, with high workloads, is associated with acute health effects. Preventive and control measures for heat stress are needed to reduce the risk of heat strain.

## 1. Introduction

Heat is a common and important physical health hazard for workers in a variety of outdoor and indoor work environments. The combination of heat exposure from the work environment (both from weather and man-made heat), and body heat generated from metabolic processes (related to workload) can cause heat gain in the body [1]. Without adequate heat dissipation, persistent high core body temperature can overwhelm the body’s thermoregulatory capabilities and result in negative health outcomes [2].

Heat related illness is among the acute negative impacts from prolonged exposure to high temperature. These effects vary from minor heat-related conditions and symptoms such as heat exhaustion, heat rash, heat edema, and heat cramps, and syncope, to major heat injuries causing death such as exertional heat injury, exertional rhabdomyolysis, and heat stroke [3]. The incidence of heart-related symptoms and illness increases when ambient temperature is higher, especially among people working in physically demanding jobs in extremely hot work environments [4]. Moreover, hot weather is positively related to occupational injuries, mostly involving increased fatigue, reduced alertness, deterioration in psychomotor abilities, and loss of concentration [5].

The impact of climate change will likely increase heat exposure and heat-related illnesses for people in equatorial climates and will mainly affect lower socio-economic status workers with manual labor jobs [6]. Thailand is among these tropical countries, with high humidity and hot weather, particular in the summer season. Data from the Thai Ministry of Natural Resources and Environment revealed that the mean temperature has risen by about 0.74 °C since the last century [7]. If this current warming trend continues, it is anticipated to increase the risk of heat related illnesses in Thailand.

The sugarcane industry is one of the major agricultural industries in Thailand. In 2015, the total sugarcane production was 105.96 million tons, cultivated on 1.62 million hectares (16.2 billion square meters) of land across the country. The total export sales of sugarcane were valued at $2708 million USD for 7.94 million tons, comprising 15% of the world sugar market [8]. Workers in this industry are exposed to various occupational hazards including heat and solar radiation during the sugarcane farming, harvesting, and manufacturing process [9]. During harvesting season, sugarcane cutters are working in the fields, exposed to outdoor extreme heat and solar radiation for long periods of time while working strenuously to manually cut and pack the cane. Unlike sugarcane cutters, factory workers are not exposed to solar radiation, but they may be exposed to high radiant heat from boilers or hot equipment used to process the sugarcane. Therefore, workers with both jobs are at risk of heat-related illness, if the preventive programs to limit heat exposures are not sufficient. Previous studies on heat stress in factory workers have focused on steel plants, glass manufacturing, foundries, and automobile industries [1]. For sugarcane workers, studies on heat stress and heat-related symptoms have been conducted in Central America. In Costa Rica, a qualitative evaluation of heat hazard in the sugarcane industry found that sugarcane workers were exposed to extreme heat in their working conditions [10]. The same research team also compared heat-related symptoms in sugarcane harvesters and workers in a sugarcane plant. The results showed that a number of heat-related symptoms were reported significantly more frequently in sugarcane harvesters than workers in the sugarcane plant [11]. In El Salvador, a study of sugarcane cutters revealed that the wet bulb globe temperature (WBGT) in the sugarcane field reached 31.2 °C, which is above the threshold limit (26 °C) for continuous work [12]. A water-rest-shade program was implemented to relieve dehydration and heat-related symptoms among the sugarcane cutters in El Salvador [13]. In Nicaragua, researchers implemented heat stress prevention measures among sugarcane cutters by providing more rehydration solutions and water during their work shift. This strategy increased productivity and awareness concerning heat stress among the cutters [14].

In Thailand, there are regulatory standards of occupational health and safety addressing heat stress for formal sector workers, but these do not apply to informal workers including seasonal sugarcane cutters. Information on heat exposure and heat-related health outcomes in the sugarcane industry of Thailand has been limited. This study focused on measuring the heat exposure, working conditions and heat-related health effects experienced by sugarcane workers during a harvest season in Nakhon Ratchasima province of Thailand. This information can be used to develop interventions and proactive approaches for reducing heat-related symptoms and illnesses in the sugarcane industry in Thailand.

## 2. Materials and Methods

### 2.1. Place, Time and Participants

The study was undertaken in Nakhon Ratchasima province in the northeastern part of Thailand, one of the largest areas for cultivation of sugarcane. In harvesting season, sugarcane at peak maturity must be cut promptly in order to maximize sugar content. To make access easier for cutters and eliminate snakes and other poisonous organisms, it is common to burn the cane fields at night before cutting the stalks. After cutting the stalks into the required lengths, they are loaded and transported to a sugar processing plant. Cane stalks are fed to roller mills for crushing the cane to extract the juice. Juice is then concentrated by vacuum boiling and evaporation process to produce crystalline sugar. In 2017, more than 7.8 million tons of sugarcane were harvested and transported to sugarcane factories in this area [15].

Participants in this study were 183 sugarcane workers working for a sugarcane company in Nakhon Ratchasima province, recruited during the harvesting season (December 2017–March 2018). The sugarcane workers were classified into two groups: 90 sugarcane cutters who were seasonal workers, and 93 sugarcane factory workers working in the milling, boiling, crystallization, and maintenance areas of the plant. All participants in the study were at least 18 years of age without a medical history of kidney disease, cardiovascular disease, diabetes, or gout. They provided informed consent to participate in this study. The study protocol was approved by the Human Research Ethics Committee, Mahidol University (MUPH 2015-146) and Suranaree University of Technology (EC-60-62).

### 2.2. Heat Stress Exposure Measurement

Environmental heat was measured using a wet bulb globe temperature (WBGT) monitor (QUESTemp 34, Quest Technologies, WI, USA) to calculate WBGT index, which integrates the effect of humidity and air movement (natural wet bulb temperature, Tnwb), globe temperature (Tg) and dry bulb air temperature (Td). WBGT outdoors was calculated for sugarcane cutters working in a field and WBGT indoors was calculated for sugarcane factory workers. The formulas are as follows:

WBGT outdoors = 0.7 Tnwb + 0.2 Tg + 0.1 Td (with direct sun exposure)

WBGT indoors = 0.7 Tnwb + 0.3 Tg (without direct sun exposure)

The WBGT measurements in both work environments were automatically recorded every 15 min for a full work shift for both cutters and factory workers. There were six monitoring stations located in the sugarcane field, and in the milling, boiling, crystallization, maintenance area, and air conditioned control room for the sugarcane factory. Monitoring at each station was carried out simultaneously. The measurements were conducted for three days, a day per week for three consecutive weeks in the hottest month (March) during the harvesting season (four months from December to March). We used the mean of these WBGTs to represent the worst case condition of workers’ heat exposure at the six work locations described above during the harvesting season. The WBGT time weighted average (TWA) incorporates the time spent in each location and is calculated as:

WBGT_TWA_ = (WBGT_1_ × Time_1_ + WBGT_2_ × Time_2_)/(Time_1_ + Time_2_).

where WBGT_1_ and WBGT_2_ represent the mean WBGT for each work location, and Time_1_ and Time_2_ represent the time spent in each work location.

The heat stress assessments were conducted based on American Conference of Governmental Industrial Hygiene (ACGIH) guidelines [16]. Workloads were also estimated through observation while WBGT measurements were being taken, following the ACGIH guidelines. The WBGT_TWA_ was compared with ACGIH threshold limit values (TLVs) for heat stress based on the intensity of the workload: 26.6 °C for heavy work, 28.2 °C for moderate work, and 30.8 °C for light work [16].

### 2.3. Physiological Measurement during Work Shift

On the last day of WBGT measurement in the workplace, blood pressure, heart rate and body temperature were measured, and urine samples collected pre- and post-shift to evaluate the effect of heat exposure during a work shift. Urine was immediately tested using a urine dipstick (CYBOW™, DFI, Gimhae, Korea) with seven parameters. The urine specific gravity (USG) was used as indicator of hydration status. USG value 1.026–1.030 indicates dehydration, and more than 1.030 indicates a clinically dehydrated state [17]. Body temperature was measured using an infrared ear thermometer (Braun GmbH, Thermoscan 7 IRT 6520, Kornberg, Germany). Blood pressure and heart rate were also measured using a sphygmomanometer (Omron, HEM-7320, Kyoto, Japan). Before these measurements, each participant took a rest for at least ten minutes. The blood pressure and heart rate measurements were measured three times with a minute interval, and then the average value was calculated. These parameters are indicators of heat strain, the body’s physiological response to heat stress.

### 2.4. Symptoms

At the end of the harvesting season, all participants were interviewed by a questionnaire including questions about demographics, previous and current work, housing, dietary habits (fluid intake, smoking and alcohol consumption), and health symptoms experienced during the harvesting season. The symptom questions consisted of 15 heat-related and eight not-heat related symptoms. Heat-related symptoms were compiled and selected from review articles on heat stress related to industrial workers [18], sugarcane harvesters [11] and other agricultural workers [19,20,21]. Non-heat related symptoms included respiratory symptoms, digestion problems, and irritation of the skin and eye. Each participant was asked whether a given symptom had occurred “regularly” (at least once per week), “sometimes” or “never” during the harvesting season. For data analysis, we grouped sometimes + regularly into an “ever” variable. This questionnaire has been previously used in other studies in Thailand [22,23,24], and it is available in the Supplementary Materials.

### 2.5. Data Analysis

Collected data were analyzed using SPSS software version 19.0 (IBM Corporation, New York, USA). Descriptive statistics were used to describe demographic characteristics, heat exposure, and health symptoms. Paired *t*-tests were used to determine differences of physiological measurement across the work shift. Chi-square test and Fisher’s exact test were used to evaluate differences in health symptoms between the sugarcane cutter and factory worker groups during the harvesting season.

## 3. Results

### 3.1. General Characteristics

The demographic data of 183 sugarcane workers is summarized in Table 1. About 58.9% of sugarcane cutters and 94.6% of factory workers were men. The mean age of cutters (42 years) was older than factory workers (39.3 years), but the difference was not statistically significant. The mean years of education of cutters (6.8 years) was about half that of factory workers, whereas the work experience of cutters (10.6 years) was two times higher than factory workers. The mean BMI of cutters (22.8) was significantly lower than factory workers (24.5). Cutters worked an average 9.4 h a day and 6.5 days a week, which was higher than factory workers (eight hours a day and six days a week). During harvesting season, more than half the cutters lived in temporary labor camps, whereas all the factory workers lived in their own houses or apartments. Alcohol drinking among cutters (41.1%) was significantly less than factory workers (72%), whereas smoking in both groups was hardly different (36.6% vs. 43.3%). Half of the cutters reported that their income was not sufficient, whereas only 18.3% of factory workers did. During harvesting season, mean fluid intake per shift of sugarcane cutters (3.8 L/shift) was significantly higher than that of factory workers (2.6 L/shift). About 44% of cutters and 69% of factory workers had fluid intake of 1–3 L/shift, whereas 6.7% of sugarcane workers drank more than 7 L/shift (Table 2).

### 3.2. WBGT Measurement in Work Environment

**Figure 1:**
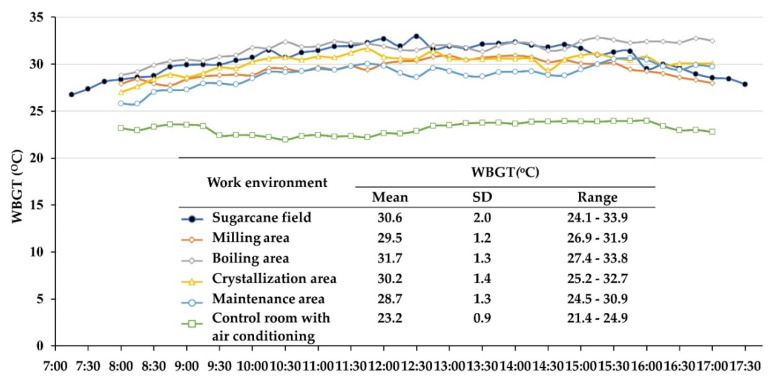
Mean of wet bulb globe temperature (WBGT) in work environments of sugarcane workers in the hottest month of the harvesting season (n = 3 measurements).

The WBGT in the hottest month of the harvesting season is shown in Figure 1. The mean WBGT in the sugarcane fields (measured from 07:30 to 17:30) was 30.6 ± 2.0 °C with a range of 24.1–33.9 °C. The WBGT in the field was low in the morning, then continuously increased until noon, remaining stable (WBGT > 30 °C) in the afternoon period and then decreasing after 15:30 p.m. For the factory, all ranges of WBGT in each workplace (measured from 08:00 to 17:00) were narrower than those of the sugarcane field. The highest mean WBGT was 31.7 ± 1.3 °C with a range of 27.4–33.8 °C in the boiling area; followed by crystallization area (30.2 ± 1.4 °C), milling area (29.5 ± 1.2 °C) and maintenance area (28.7 ± 1.3 °C). The lowest mean WBGT was 23.2 ± 0.9 °C in the control room with air conditioners.

### 3.3. Heat Stress Assessment

The workloads and heat exposures of sugarcane workers are illustrated in Table 3. The sugar cutter job was classified as heavy work (415 watts), whereas for factory workers, their jobs varied from moderate work (300 watts) for the maintenance job to light work (180 watts) for the milling, boiling and crystallization jobs. After estimating WBGT_TWA_ we compared heat exposure with the ACGIH heat stress TLVs based on the intensity of workload. We found that sugarcane cutters were at high risk of heat stress (WBGT > 26.6 °C TLV for heavy work). However, low risk of heat stress was found in all sugarcane factory jobs including milling, boiling and crystallization workers (WBGT < 30.8 °C TLV for light work), and maintenance workers (WBGT < 28.2 °C TLV for moderate work).

### 3.4. Physiological Measurement during Work Shift

Blood pressure, heart rate and temperature across the work shift among sugarcane workers are shown in Table 4. All values, except the diastolic blood pressure (DBP) of factory workers, increased at post-shift. However, only in cutters were these differences significant. For cutters, the mean increase of systolic blood pressure (SBP), heart rate, left and right ear temperature was 3.2 mmHg, 8.5 bpm, 0.6 °C, and 0.5 °C, respectively. 

The results of urine tests across the work shift among sugarcane workers are shown in Table 5. The percentage of workers with urine specific gravity (USG) at 1.030 (dehydrated) significantly increased from 16.7% at pre-shift to 53.3% at post-shift in cutters, but only from 12.9% to 15.1% in factory workers. Workers in both groups were much more likely to have acidic urine (pH 5–6) at pre- and post-shift (about 70% in cutters and 50% in workers). For blood, glucose, protein, nitrite and leucocytes in urine, the pre- and post-shift tests showed a high percentages of negative test results. However, leucocytes were detected with a 9% cross shift increase in the cutter group.

### 3.5. Prevalence of Heat-Related Symptoms

During the harvesting season, the number of sugarcane workers experiencing symptoms is described in Table 6. The common heat-related symptoms (>50%) reported by cutters were weakness/fatigue (91.1%), heavy sweating (83.3%), headache (57.8%), rash (52.2%), and muscle cramp (52.2%). Factory workers commonly reported weakness/fatigue (64.5%) and heavy sweating (52.7%). A comparison between cutters and factory workers, for heat-related symptoms “ever” experienced found cutters had significantly more symptoms of weakness/fatigue, heavy sweating, headache, skin rash, muscle cramps, dry mouth, dizziness, dry/cracking skin, swelling of hands/feet, skin blisters, and fainting. These differences were largely the same when analyzed using “regularly” (at least one per week) experienced symptoms, with the exception of skin blisters and fainting, whereas fever occurred in the cutters significantly higher than in factory workers only when analyzed using “regularly”. The top three “regular” symptoms that were significantly higher in cutters compared with factory workers were heavy sweating (60.0% vs. 18.3%), weakness/fatigue (31.1% vs. 8.6%), and dry mouth (15.6% vs. 5.4%). Fever (6.7%), dry/cracking skin (6.7%), swelling of hands/feet (5.6%), and dizziness (5.6%) were only regularly reported in the cutter group. For non-heat related symptoms, eye irritation and itchy skin were reported significantly more frequently by sugar cane cutters both “ever” and “regularly. Nose congestion and cough were reported as regular symptoms more significantly among sugarcane cutters.

## 4. Discussion

Sugarcane cutters are a seasonal temporary workforce, part of the informal work sector in Thailand. The majority are farmers from the northeast area of Thailand with only a primary education. When the rice harvesting season ends in one area, they migrate to other areas to work as seasonal sugarcane cutters. Although sugarcane cutting is physically demanding work [25,26,27], we found the proportion of women working in this occupation was about 40%, which is consistent with a study of sugarcane cutters in Cambodia [28]. This situation may be caused by more recent economic factors that drive rural men to move to urban areas to work in the industrial sector or in other higher paying jobs [22]. The wages of sugarcane cutters depend on how much they cut, mostly 0.5–0.7 Baht (~US Cent 1.7–2.3)/binder for burnt cane (10–16 stalks) and 1.5–2 baht (~US Cent 5.0–6.7)/binder for green cane [29]. Due to the short harvesting season, cutters try to work as much as they can to get the maximum income provided by this seasonal work [30]. In this study the cutters worked on average about 9.4 h/day and 6.5 days/week. However, we found that 50% reported that their income was not sufficient. Most cutters live in transient labor camps with poor living conditions that increase their vulnerability.

Unlike sugarcane cutters, process workers in sugarcane factories are permanent workers. Most of them are men who have graduated from high school or have a vocational certificate. The wage paid depends on the workers’ skill and job position; however, they receive at least 300 baht/day (~US $10), the minimum wage set by Thai government. Despite the peak production associated with the harvesting season, workers are not allowed to work over 48 h/week and they are required to have at least one day off/week. All workers live in their own house or apartment. About 80% of the workers reported their income was sufficient and 44% of them had savings. However, the mean BMI and the percentage drinking alcohol among factory workers were higher than among cutters, which may be associated with the gender divide between these job groups.

During the harvesting season, we found that cutting sugarcane is heavy work, whereas the sugarcane factory process tasks are of light to medium. Considering only WBGT in the workplaces, the mean value in the boiling area (31.7 °C) is higher than in the sugarcane field (30.6 °C). However, workers with a boiling job are at lower risk of heat stress than sugarcane cutters due to the combination of workload, WBGT in workplace, and the time duration workers are exposed to heat each workday. Sugarcane cutters work most of the day (8–11 h) with intensive physical activity outdoors and usually take a rest under trees or in the shade of the sugar cane so they are still exposed to the heat. In addition to heat exposure from weather conditions, heat from the burning field the night before cutting could increase heat stress among sugarcane cutters [10]. Because the WBGT (30.6 °C) that the cutters are exposed to is over the recommended TLV of 26.6 °C based on workload, they are at high risk of heat stress. Unlike sugarcane cutters, factory workers are at low risk of heat stress, because they are not working in a hot workplace for all their work time; for example, workers in the boiling section work in hot areas for only 30% of the workday, and spend 70% of their workday in the control room with air conditioning. The cooling system and air ventilation in the indoor workplace can reduce heat exposure among indoor workers [31]. In a review of 43 articles by Xiang, et.al. [1], about 79% of epidemiological studies reported that 90% of participants were suffering from heat stress during outdoor work compared to those who were indoor worker (65%). Moreover, many studies report that, when heat preventive measures are not adequately implemented, outdoor workers under the sun with heavy workloads are susceptible to heat stress [4,10,32,33,34]. Our findings are consistent with studies that reported sugarcane cutters are at high risk for exposure to heat stress [14,25], whereas sugarcane process workers are at low risk [35].

Across the work shift, we found that a half of the sugarcane cutters were dehydrated by the end of their work shift, indicating that their mean fluid intake per shift (3.8 L) was not sufficient for adequate hydration to maintain a stable body core temperature. Additionally, we found a high percentage of low urine pH among the cutter group. This acidification of urine could result from the lactate generated by muscle, the effect of aldosterone [36], and volume (salt) depletion [37]. This aligns with the studies of sugarcane cutters in El Salvador [36], Nicaragua [38], and Costa Rica [39] which reported that heat stress, dehydration and lower urinary pH were most common among sugarcane cutters. Additionally, the study in Nicaragua also revealed that sugarcane cutters more often had blood in their urine, leucocytes and proteinuria compared to constructions and farming workers [38]. Similarly, we found that 13% of cutters had blood in their urine, and at post-shift the percentage of cutters with leucocytes in their urine significantly increased by about 9%. Urinary acidification across a working day suggests insufficient hydration, and positive blood on the dipstick may be a sign of mild rhabdomyolysis [39]. According to the US National Institute of Occupational Safety and Health (NIOSH) guidelines, working under heat stress conditions can result in the loss of body water via sweating, reaching 6–8 L a working shift, so this needs to be replaced by drinking water or other fluids every 15–20 min. For highly intensive work in a hot environment for ≥2 h, sport drinks are recommended to replace the electrolytes lost from sweating and to avoid hyponatremia [40]. The California Occupational Safety and Health Association (OSHA) requires a 240 mL cup of water every 15 min (7.7 L/8 h-shift) for working outdoor in extreme heat condition [41].

Among sugarcane cutters with high risk of heat stress, we found a significant increase in heart rate, temperature, and blood pressure (SBP) occurred across the work shift. An experimental study showed a significant increase in heart rate (+20.2 bpm) and ear temperature (+0.9 °C) occurred after subjects were exposed to hot conditions (WBGT = 35 °C), but not for those in a thermoneutral environment (WBGT = 21 °C) [42]. Parasympathetic withdrawal may be a cause of the increase in heart rate observed in hot conditions [43]. For sugarcane cutters, a study in Cambodia reported significant physiological increases (heart rate, ear temperature, SBP, and DBP) during working compared with a resting period [28]; however, a study in El Salvador found a significant increase in heat rate (+17 bpm), but a decrease of SBP (−8 mmHg) and DBP (−5 mmHg) across 4-h shift [36].

Heat-related symptoms are often reported by workers working in hot workplaces including outdoor environments. The vast majority of Latin migrant farm workers in North Carolina worked in an outdoor extreme heat environment, and nearly half experienced heat-related symptoms. The incidence of symptoms increased in higher heat environments (4). About two-thirds of migrant farm workers in Oregon reported heat related symptoms, most commonly heavy sweating (50%) and headache (24%) [20]. For sugarcane workers, a study of 110 cutters in Cambodia revealed that most cutters experienced heavy sweating (87.2%) and tiredness/weakness (86.4%). Their prevalence of headache and muscle cramps was about 60%, and 41% for dizziness [28]. Since heat-related symptoms are common and may be due to other factors, a study in Costa Rica [11] focused on the frequency of heat-related symptoms (occurring at least once a week) among 106 sugarcane harvesters compared to 63 non-heat exposed workers in a sugarcane company that included processing plant and mechanic shop workers. Their results showed that headache, tachycardia, muscle cramps, fever, nausea, difficult breathing, dizziness, and swelling hands/feet were symptoms that sugarcane harvesters reported significantly more frequently than non-harvesters, including processing plant and mechanic shop workers. This suggests that the symptoms could be a result of heat exposure. Their findings were consistent with our study, with the exception of tachycardia and difficulty breathing. In addition to heat-related symptoms, respiratory symptoms (nose congestion and cough) and irritation of eye and skin were regular symptoms more significantly found among the sugarcane cutters in our study. The respiratory symptoms were also reported in other sugarcane cutter groups [11,44]. Pre-harvest burning and ash that may be easily broken down into particulate matter during cutting sugarcane in burnt fields can be potential sources of airborne particulate that may cause both acute and chronic respiratory health effect [45], and irritation of eye and skin.

There are some limitations to this study. The gender proportions among the sugarcane cutters compared to the factory workers are different and could impact comparisons. However, this is the real situation in Thailand. Symptoms were recalled by self-report at the end of the harvesting season, so may be subject to recall bias. The healthy worker effect may occur among factory workers, who work in this job all year, compared to the migrant seasonal sugarcane cutter population. WBGT measurements were only made once a week for three consecutive weeks to represent the whole harvest season, and workload was determined by observation without participation of the workers. Physiological measurements were conducted pre- and post-work shift, not across the whole day. However, since the WBGT and physiological measurements were collected in a single situation, the hottest month of the harvesting season, we believe these data represent the worst case condition of workers’ heat stress and physiological strain in the harvesting season. Finally, the study does not report on potential chronic cardiovascular [46] and kidney disease risk [27,35,36,39] in this population.

### Implication for Protection of Informal and Seasonal Sugarcane Cutters

Unlike formal workers, most of the Thai labor laws and regulations do not apply to informal workers because they lack employment contracts. Since 2011, the Thai Social Security Act has allowed informal workers to be voluntarily insured, but they must pay 100–150 baht/month (~US $3–5). This is considered too expensive by most informal workers, like sugarcane cutters, so few obtain benefits from the Act [47]. In addition, Thailand lacks legally mandated occupational health and safety programs for informal workers, therefore there are currently no heat stress and heat-related illness regulations or prevention programs that apply to this workforce.

For formal sector workers, the law requires employers to control and maintain the WBGT in the workplace so as not to exceed 30 °C, 32 °C, and 34 °C for heavy, moderate, and light work, respectively [48], and to provide their employees with annual health checkups based on job risks and exposures [49]. In addition, the employer must hire a professional safety officer to inspect and assess risks in the workplace and give recommendations to the employer to comply with the occupational safety, health and environmental regulations [50]. This may, in part, explain why plant workers in sugarcane factories were at low risk of heat stress.

Based on the findings in this study, sugarcane cutters in Thailand experience high risk of heat stress and commonly reported heat-related symptoms during the harvesting season. A number of these heat-related symptoms were reported significantly more frequently in sugarcane cutters than among the sugarcane factory workers with low risk of heat stress. Dehydration and physiological effects of heat exposure across the work shift were observed among the sugarcane cutters. Implementation of interventions to prevent and reduce heat stress among sugarcane cutters are recommended. Firstly, a water-rest-shade (WRS) program should be implemented. Bodin et al., (2016) demonstrated that a WRS program providing appropriate access to shade (a portable canopy), rest (10–15 min break/1–1.5 h), and water (25% increase of water consumption), relieving dehydration, heat-related symptoms and health problem among sugarcane cutters in El Salvador [13]. Moreover, studies reported that the program improved cutters’ productivity [13,14,51]. Secondly, education and training on heat-illness prevention should be provided to sugarcane cutters and foremen. The local primary health care unit (PCU)/health promoting hospital should promote and train workers to be aware of and familiar with the early signs and symptoms of heat-related illness. Preventive action could prevent a heat-induced physiological reaction that could result in critical illness and death [52]. This education and training can reduce occupational heat stress and illness, without decreasing workers’ performance and productivity, and is essential for effective management of heat stress risks [53]. Due to the heavy physical demand of sugarcane manual cutting, use of cutting machines is a good option to minimize workload and the impact of heat exposure. In addition to health benefits, this is a good solution for current labor shortages, and the environmental impacts from burning sugarcane, which is presently an important problem for the sugarcane industry. Finally, more research studies are needed to develop and test appropriate interventions and preventive approaches for sustainable solutions to heat-related illness, especially among informal laborers who are not covered by occupational health and safety laws and regulations. For example, a customized heat warning system, the HEAT-SHIELD Platform, was developed in Europe to provide a forecast of the daily risk of heat stress and behavioral suggestions to reduce hazard, such as hydration and altered work-rest patterns. This system could support the planning of activities in various occupational contexts [54].

## 5. Conclusions

During the harvesting season in Thailand, sugarcane cutters are at risk of heat stress and strain because of their physically demanding jobs in an extreme equatorial work environment. Measurements of heat stress and strain were conducted during the hottest month (March) of the harvest season in Thailand. Based on measurements and observations taken one day per week over three consecutive weeks in March, sugarcane cutters’ heat exposure exceeded the ACGIH TLV for heat stress, while that of sugarcane factory workers did not. Significant cross shift physiological effects from heat exposure were observed and a high prevalence of heat-related symptoms were reported among sugarcane cutters, compared with sugarcane factory workers. These findings raise the need for government agencies, the sugarcane industry, and researchers to develop strategic plans, and preventive approaches, to reduce the acute and potentially chronic health effects from occupational heat stress, especially among seasonal temporary laborers who lack specific government regulations or enforcement mechanisms to prevent and control the problem.

## Figures and Tables

**Table 1:** Characteristics of study subjects.

Characteristic	Cutter (n = 90)	Factory Worker (n = 93)	*p*-Value
Male gender, n (%)	53 (58.9)	88 (94.6)	<0.001 ^a^
Age (year), Mean ± SD (range)	42.0 ± 11.0 (18–60)	39.3 ± 7.5 (21–54)	0.052 ^b^
Education (years) Mean ± SD (range)	6.8 ± 1.7 (6–14)	12.0 ± 2.9 (6–16)	<0.001 ^b^
Work experience (year), Mean ± SD (range)	10.6 ± 9.4 (1–40)	5.6 ± 5.1 (1–29)	<0.001 ^b^
BMI, Mean ± SD (range)	22.8 ± 4.2 (15.5–34.7)	24.5 ± 4.0 (17.1–35.6)	0.005 ^b^
Workdays per week, Mean ± SD (range)	6.5 ± 0.4 (6–7)	6.0 ± 0.0 (6)	<0.001 ^b^
Work hours per day, Mean ± SD (range)	9.4 ± 0.87 (8–11)	8.0 ± 0.3 (8–10)	<0.001 ^b^
Housing: labor camp, n (%)	56 (62.2)	0 (0.0)	<0.001 ^a^
Drink alcohol, n (%)	37 (41.1)	67 (72.0)	<0.001 ^a^
Smoking, n (%)	39 (43.3)	34 (36.6)	0.369 ^a^
Income			
Sufficient with saving, n (%)	11 (12.2)	41 (44.1)	<0.001 ^a^
Sufficient without saving, n (%)	34 (37.8)	35 (37.6)	
Not sufficient with debt, n (%)	45 (50.0)	17 (18.3)	

^a^*p*-Value from Chi-square test; ^b^ Independent *t*-test.

**Table 2:** Fluid intake per shift of sugarcane workers (90 cutters and 93 factory workers).

Fluid Intake (L/shift)	Cutter (n, (%))	Factory Worker (n, (%))	*p*–Value
1.1–3.0	40 (44.4)	64 (68.8)	<0.001 ^a^
3.1–5.0	28 (31.1)	27 (29.0)	
5.1–7.0	16 (17.8)	1 (1.1)	
> 7.0	6 (6.7)	1 (1.1)	
Mean ± SD (range)	3.8 ± 2.0 (1.1–11.1)	2.6 ± 1.2 (1.0–7.8)	<0.001 ^b^

^a^*p*-Value from Fisher’s exact test; ^b^ Independent *t*-test.

**Table 3:** Classification of workload and heat exposure for sugarcane workers.

Job	Tasks Observed (% ^a^)	Workload Classified	Heat stress ^b^ (WBGT_TWA_, °C)
Cutters (n = 90)	Cut cane using machete, strip the leaves off, and pile stalk in rows on the ground (100%)	Heavy	30.6
Factory workers (n = 93)			
Milling	Oversee milling operation in control room with air conditioning (70%), check and conduct quality control procedure in milling area (30%)	Light	25.1
Boiling	Oversee boiling operation in control room with air conditioning (70%), check and conduct quality control procedure in boiling area (30%)	Light	25.8
Crystallization	Oversee crystallization process in control room with air conditioning (70%), check and conduct quality control procedure in crystallization area (30%)	Light	25.3
Maintenance	Maintain machine and equipment in maintenance shop (80), and work in air-conditioned room (20%)	Moderate	27.6

^a^ percent of working time; ^b^ WBGT_TWA_ = (WBGT_1_ × Time_1_ + WBGT_2_ × Time_2_)/(Time_1_ + Time_2_), when WBGT_1_ and WBGT_2_ derived from the mean of WBGT in each workplace (Figure 1).

**Table 4:** Blood pressure (BP), heart rate, and ear temperature of sugarcane workers in the work shift.

Parameter	Pre-Shift	Post-Shift	Difference	*p*-Value ^a^
	Mean (SD)	Mean (SD)		
Systolic BP (mmHg)				
Cutter (n = 90)	122.8 (11.8)	126.0 (12.4)	3.2	<0.001
Factory worker (n = 93)	125.7 (13.8)	126.2 (12.1)	0.5	0.680
Diastolic BP (mmHg)				
Cutter (n = 90)	76.7 (8.7)	77.8 (9.2)	1.1	0.185
Factory worker (n = 93)	80.6 (9.4)	80.6 (10.3)	0.0	0.942
Heart rate (bpm)				
Cutter (n = 90)	75.9 (11.3)	84.4 (13.2)	8.5	<0.001
Factory worker (n = 93)	77.3 (13.3)	81.0 (15.3)	3.7	0.003
Left ear temperature (°C)				
Cutter (n = 90)	36.5 (0.5)	37.1 (0.5)	0.6	<0.001
Factory worker (n = 93)	36.7 (0.4)	36.8 (0.4)	0.1	0.569
Right ear temperature (°C)				
Cutter (n = 90)	36.6 (0.5)	37.1 (0.5)	0.5	<0.001
Factory worker (n = 93)	36.7 (0.4)	36.8 (0.4)	0.1	0.693

^a^*p*-Value from paired *t*-test.

**Table 5:** Urine dipstick measures among sugarcane workers across the work shift.

Parameter	Cutter (n = 90)		*p*-Value ^a^	Factory Worker (n = 93)		*p*-Value ^a^
	Pre-Shift n (%)	Post-Shift n (%)		Pre-Shift n (%)	Post-Shift n (%)	
Urine SG						
≤1.010	14 (15.6)	11 (12.2)	<0.001	27 (29.0)	21 (22.6)	0.664
1.020	33 (36.7)	14 (15.6)		27 (29.0)	25 (26.9)	
1.025	28 (31.1)	17 (18.9)		27 (29.0)	33 (35.5)	
1.030	15 (16.7)	48 (53.3)		12 (12.9)	14 (15.1)	
pH						
5–6	63 (70.0)	64 (71.1)	0.612	53 (57.0)	51 (54.8)	0.296
6.5–7	20 (22.2)	16 (17.8)		31 (33.3)	26 (28.0)	
8–9	7 (7.8)	10 (11.1)		9 (9.7)	16 (17.2)	
Blood						
Negative	79 (87.8)	78 (86.7)	0.823	83 (89.2)	84 (90.3)	0.809
Positive	11 (12.2)	12 (13.3)		10 (10.8)	9 (9.7)	
Glucose						
Negative	87 (96.7)	89 (98.9)	0.621 ^b^	92 (98.9)	92 (98.9)	1.000 ^b^
Positive	3 (3.3)	1 (1.1)		1 (1.1)	1 (1.1)	
Protein						
Negative	87 (96.7)	85 (94.4)	0.720 ^b^	88 (94.6)	86 (92.5)	0.551
Positive	3 (3.3)	5 (5.6)		5 (5.4)	7 (7.5)	
Nitrite						
Negative	90 (100.0)	89 (98.9)	1.000 ^b^	92 (98.9)	93 (100.0)	1.000 ^b^
Positive	0 (0.0)	1 (1.1)		1 (1.1)	0 (0.0)	
Leucocytes						
Negative	87 (96.7)	79 (87.8)	0.026	89 (95.7)	91 (97.8)	0.682 ^b^
Positive	3 (3.3)	11 (12.2)		4 (4.3)	2 (2.2)	

^a^*p*-Value from Chi-square test; ^b^ Fisher’s exact test.

**Table 6:** Prevalence of symptoms reported by sugarcane worker during the harvesting season.

Symptom	Ever		*p*-Value ^a^	Regularly		*p*-Value ^a^
	Cutter	Worker		Cutter	Worker	
	n (%)	n (%)		n (%)	n (%)	
**Heat-related symptom**						
Weakness/fatigue	82 (91.1)	60 (64.5)	<0.001	28 (31.1)	8 (8.6)	<0.001
Heavy sweating	75 (83.3)	49 (52.7)	<0.001	54 (60.0)	17 (18.3)	<0.001
Headache	52 (57.8)	34 (36.6)	0.004	11 (12.2)	2 (2.2)	0.008
Rashes on skin	47 (52.2)	19 (20.4)	<0.001	14 (15.6)	2 (2.2)	0.001
Muscle cramps	47 (52.2)	22 (23.7)	<0.001	12 (13.3)	2 (2.2)	0.004
Dry mouth	41 (45.6)	29 (31.2)	0.045	14 (15.6)	5 (5.4)	0.024
Dizziness	36 (40.0)	18 (19.4)	0.002	5 (5.6)	0 (0.0)	0.027 ^b^
Fever	30 (33.3)	28 (30.1)	0.639	6 (6.7)	0 (0.0)	0.013 ^b^
Dry, cracking skin	22 (24.4)	8 (8.6)	0.004	6 (6.7)	0 (0.0)	0.013 ^b^
Swelling hands/feet	15 (16.7)	1 (1.1)	<0.001	5 (5.6)	0 (0.0)	0.027 ^b^
Blisters on skin	21 (23.3)	8 (8.6)	0.006	3 (3.3)	1 (1.1)	0.363 ^b^
Fainting	7 (7.8)	1 (1.1)	0.033 ^b^	2 (2.2)	0 (0.0)	0.240 ^b^
Nausea	12 (13.3)	9 (9.7)	0.438	3 (3.3)	0 (0.0)	0.117 ^b^
Dysuria	5 (5.6)	1 (1.1)	0.114 ^b^	0 (0.0)	0 (0.0)	1.000 ^b^
Vomiting	4 (4.4)	3 (3.2)	0.718 ^b^	2 (2.2)	0 (0.0)	0.240 ^b^
**Non-heat related symptom**						
Eye irritation	58 (64.4)	36 (38.7)	<0.001	12 (13.3)	2 (2.2)	0.004
Itchy skin	54 (60.0)	31 (33.3)	<0.001	19 (21.1)	3 (3.2)	<0.001
Nose congestion	49 (54.4)	45 (48.4)	0.412	15 (16.7)	5 (5.4)	0.014
Cough	44 (48.9)	41 (44.1)	0.515	12 (13.3)	3 (3.2)	0.013
Loss of appetite	33 (36.7)	7 (7.5)	<0.001	3 (3.3)	0 (0.0)	0.117 ^b^
Chest tightness	14 (15.6)	10 (10.8)	0.336	2 (2.2)	0 (0.0)	0.240 ^b^
Wheezing	9 (10.0)	4 (4.3)	0.134	2 (2.2)	0 (0.0)	0.240 ^b^
Digestive problems	27 (30.0)	22 (23.7)	0.333	6 (6.7)	3 (3.2)	0.325 ^b^

^a^*p*-Value from Chi-square test; ^b^ Fisher’s exact test.

## Supplementary Materials

The following are available online at https://www.mdpi.com/1660-4601/17/17/6363/s1, Questionnaire.
