# Supplementary material for: Heat Stress, Physiological Response, and Heat-Related Symptoms among Thai Sugarcane Workers
Source: Int J Environ Res Public Health. 2020 Sep 1;17(17):6363. doi: 10.3390/ijerph17176363 (PMC7503547; doi:10.3390/ijerph17176363)
Supplement: Supplementary file 1 [file ijerph-17-06363-s001.pdf]

Supplementary material

Questionnaire

Heat-related symptoms among Thai sugarcane workers

Sample ID: ………. Date of interview: …………………… Interviewer Code: ………

Please put ✓ in answer box and fill in information about you.

Section I: Personal information

1. Gender ( ) male ( ) female
2. Age .... years
3. Highest education completed (education years)
   ( ) no education (0 years) ( ) primary school (6 years)
   ( ) secondary school (9 years) ( ) high school (12 years)
   ( ) technical school (12 year) ( ) higher technical school (14 years)
   ( ) associates degree college (14 years) ( ) bachelors degree (16 years)
   ( ) more than bachelors degree (more than 16 years, specify……years)
   ( ) other, please specify……………………………………………..
4. Marital status
   ( ) single ( ) married
   ( ) widowed ( ) divorced
5. Body weight ………………… kilograms
6. Height ………… centimeters
7. Housing during the harvesting season
   ( ) camp
   ( ) house
   ( ) apartment
   ( ) other, please specify……………………………………………
8. Level of income sufficiency
   ( ) sufficient with savings
   ( ) sufficient without savings
   ( ) not sufficient with debt
   ( ) refused to answer
9. What is your job?
   ( ) sugarcane worker
   ( ) sugarcane factory worker (if choose this answer, please specify your task)
      ( ) milling
      ( ) boiling
      ( ) crystallization
      ( ) maintenance
      ( ) other, please specify……………………………………………
10. How many years have you worked for your job? ……. years
11. Average work duration........................days per week, and ………..hours per day
12. When did you start working? ………………..
13. How many total minutes of break do you get in the morning? ……………
14. How many total minutes of break do you get in the afternoon? ………….
15. When do you stop working?...........................

Section II: Health behavior.

1. Smoke
   ( ) No (go to 2) ( ) Yes (go to 1.1) ( ) Used to smoke (go to 1.2 and 1.3)

1.1 (   ) if yes, cigarrettes per day…….. How long..............................years
1.2 (   ) how long smoke till stop? ....yearss
1.3 how long ago did you stop? ……………. years

2. Ever drink alcohol
(   ) never (go to Section III)   (   ) yes   (   ) Used to drink
2.1 if yes, Duration of drinking ....years
2.2 if yes, frequency (   ) every day (   ) 2-3 times per week ( ) once a month (   ) occasionally
2.3 if used to drink how long drink till stop? ....years.
2.4. how long ago did you stop? ....years.

<u>Section III: Health symptoms</u>

In the past harvesting season (4 months), have you had any of these health problems?
Please put ✓ in the answer box.

Symptom	Answer		
	Regularly	Sometimes	Never
1. Muscle cramps			
2. Weakness/fatigue			
3. Heavy sweating			
4. Fainting			
5. Dry mouth			
6. Rashes on skin			
7. Dry, cracking skin			
8. Blisters on skin			
9. Swelling hands/feet			
10. Headache			
11. Fever			
12. Dizziness			
13. Nausea			
14. Dysuria			
15. Vomiting			
16. Itchy skin			
17. Eye irritation			
18. Nose congestion			
19. Cough			
20. Chest tightness			
21. Wheezing			
22. Loss of appetite			
23. Other abnormal symptom<br>Please specify……………………………………			

Section IV: Fluid intake

During the harvest season how much do you drink (ml per day)? (train interviewer to calculate based on answer, or could have coded pictures)

Time	Normal temp. water	Ice water	Mineral water	Carbonated water	Sweet water	Alcohol	Energy drink
Breakfast							
Morning work time							
Lunch							
Afternoon work time							
Dinner until bed							
